# Optimizing deep neural networks for high-resolution land cover classification through data augmentation

**DOI:** 10.1007/s10661-025-13870-5

**Published:** 2025-03-18

**Authors:** Sergio Sierra, Rubén Ramo, Marc Padilla, Adolfo Cobo

**Affiliations:** 1 Complutum Tecnologías de la Información Geográfica, COMPLUTIG, 28801 Alcalá de Henares, Spain; 2 Photonics Engineering Group, Universidad de Cantabria, 39005 Santander, Spain; 3 Instituto de Investigación Sanitaria Valdecilla (IDIVAL), 39011 Santander, Spain; 4 CIBER de Bioingeniería, Biomateriales y Nanomedicina (CIBER-BBN), Instituto de Salud Carlos III, 28029 Madrid, Spain

**Keywords:** Land cover classification, Data augmentation, Deep learning, Image segmentation

## Abstract

This study presents an innovative approach to high-resolution land cover classification using deep learning, tackling the challenge of working with an exceptionally small dataset. Manual annotation of land cover data is both time-consuming and labor-intensive, making data augmentation crucial for enhancing model performance. While data augmentation is a well-established technique, there has not been a comprehensive and comparative evaluation of a wide range of data augmentation methods specifically applied to land cover classification until now. Our work fills this gap by systematically testing eight different data augmentation techniques across four neural networks (U-Net, DeepLabv3 + , FCN, PSPNet) using 25 cm resolution images from Cantabria, Spain. In total, we generated 19 distinct training sets and trained and validated 72 models. The results show that data augmentation can boost model performance by up to 30%. The best model (DeepLabV3 + with flip, contrast, and brightness adjustments) achieved an accuracy of 0.89 and an IoU of 0.78. Additionally, we utilized this optimized model to generate land cover maps for the years 2014, 2017, and 2019, validated at 580 samples selected based on a stratified sampling approach using CORINE Land Cover data, achieving an accuracy of 87.2%. This study not only provides a systematic ranking of data augmentation techniques for land cover classification but also offers a practical framework to help future researchers save time by identifying the most effective augmentation strategies for this specific task.

## Introduction

Knowing the distribution and changes in land cover is essential for understanding environmental processes (Da Ponte et al., 2017), assessing habitat quality, identifying risk areas (Boori et al., 2021), monitoring deforestation (Weiland et al., 2021), and planning sustainable development (Turner, 2010). Understanding land cover data is not only essential for environmental monitoring and sustainable development but also plays a crucial role in studies such as hydrological processes: infiltration, runoff, and evapotranspiration, as highlighted by Beven and Kirkby (1979). Urbanization, for instance, can alter hydrological responses, with studies like Oudin et al. (2018) demonstrating the impact of changes in urban land cover on hydrological systems. More recently, research by Song et al. (2022) and Huynh et al. (2024) has explored the use of land cover data in regionalized hydrological modeling, providing advanced tools for predicting hydrological dynamics under various scenarios. These studies underscore the growing importance of accurate and detailed land cover information for understanding and managing hydrological systems, further validating the need for advanced methods such as those explored in this work. Land cover changes also influence ecosystem services, including water purification, carbon sequestration, and habitat provision. For instance, Makwinja et al. (2021) explore how land use and land cover dynamics impact ecosystem service value in the Lake Malombe area, Southern Malawi, highlighting the importance of monitoring these changes to ensure the sustainability of ecosystem functions. In addition to the hydrological processes, land cover plays a crucial role in water quality, with remote sensing providing valuable insights into this relationship. Gani et al. (2023) assess the impact of land use and land cover on river water quality using remote sensing techniques and water quality indices, demonstrating how changes in land cover can significantly affect water pollution levels and river ecosystems. The range of applications for remote sensing is extensive, and it has been boosted in recent years by the availability of open data and the possibilities offered by cloud computing services such as Google Earth Engine or CREODIAS. These services provide users with a wide range of image catalogues and resources for processing them and quickly obtaining results, leading to the development of a large number of scientific studies and industrial applications.

Among the most relevant applications of remote sensing, the use of time series of images for vegetation monitoring stands out. The cartography of land cover and its changes is fundamental in land management and natural resources. Land cover (LC) maps can be used for different applications, such as crop mapping, identification and monitoring of vegetation formations (Xie et al., 2008), or planning urban growth (Akbari et al., 2003).

Traditional methods for obtaining information on land cover involved the visual interpretation of aerial photographs and the digitisation of the different elements present in them. For example, the CORINE (Coordination of Information on the Environment) project (Büttner et al., 2004) and the SIOSE (Information System on Land Occupation in Spain) project (Bosque González et al., 2005) used visual interpretation and digitisation to obtain detailed maps of land cover in Europe and Spain, respectively. However, these traditional methods have limitations in terms of scalability and efficiency. Visually interpreting large volumes of data can be laborious and subjective, and manual digitisation can be a slow and laborious process. For these reasons, implementing more automated and efficient approaches is sought.

In this context, supervised classifications have gained popularity in remote sensing for obtaining information on land cover (Alem & Kumar, 2020; Sefrin et al., 2020). These methods use machine learning algorithms to automatically assign the different land cover categories based on the spectral characteristics of satellite images (Marmanis et al., 2015; Vali et al., 2020). Supervised classification algorithms are trained using labelled samples of different land cover classes and then applied to unlabelled images to perform the classification. The use of machine learning in supervised classification has proven effective for land cover segmentation tasks due to its ability to process large volumes of data and recognize complex patterns in the spectra of images at different scales (Abdali et al., 2024; Cuypers et al., 2023; Marmanis et al., 2015; Sefrin et al., 2020). This has led to an improvement in the accuracy and efficiency of obtaining information on land cover compared to traditional methods (Cuypers et al., 2023).

On the other hand, deep learning (DL), a branch of artificial intelligence, has become a powerful tool for processing complex data and extracting patterns. The integration of remote sensing and deep learning has led to significant advances in the ability to analyze and understand data collected by remote sensors. This combination allows automating tasks that previously required significant manual intervention and provides the possibility of extracting detailed information from large remote sensing datasets. The deep learning algorithm is an automatic model that refers to the ability of multi-layer neural networks to learn and recognize complex patterns and representations of datasets (Goodfellow et al., 2016; LeCun et al., 2015). Unlike traditional machine learning approaches, DL has proven to be especially effective in image processing (Krizhevsky et al., 2012), speech recognition, text analysis, and other high-level domains. When applied to the field of remote sensing and satellite images, computer vision algorithms based on DL offer great potential in the realm of LC (Alem & Kumar, 2020).

Using deep neural networks, such as Convolutional Neural Networks (CNNs), promising results have been obtained in the classification of different types of land cover, such as vegetation, water bodies, and urban areas, among others. These DL-based methods have shown a greater ability to capture complex spatial and spectral features of satellite images, leading to improved accuracy in classification (Naushad et al., 2021).

Training a deep learning model can meet considerable challenges, whether due to the vast amount of data required or the high computational requirements involved. The process of manually labelling data, a complex task, demands a substantial part of the time dedicated to the production and elaboration of maps. For this reason, various studies have opted to leverage existing cartography, such as that provided by the CORINE Land Cover (Büttner et al., 2004), as a resource to support the generation of reference data in the creation of new products or the training of classification algorithms.

For this reason, the use of data augmentation in training models with deep learning is very common (Hao et al., 2023; Imbert, 2019). The core principle of this method lies in applying transformations to previously labelled images using techniques that modify their colour, geometry, or both simultaneously, generating a more diverse dataset through synthetic images that are similar yet distinct from the originals. This process enhances the generalization capacity of models and improves their adaptability to environmental complexities, providing a robust foundation for land cover mapping. By manually labelling a small portion of data and applying various image processing techniques, new synthetic data can be created to enrich the variability of training datasets and address class imbalances. In the context of land cover classification, data augmentation strategies such as rotation, flipping, cropping, translation, and adding noise have been widely used to enhance the performance of deep learning models, demonstrating their effectiveness in improving model generalization and accuracy (Du et al., 2021).

Given the high complexity and computational cost of generating a neural network, one of the most adopted techniques is transfer learning. This allows the leveraging of knowledge previously gained by a neural network trained on a large and diverse dataset and the transfer of that knowledge to a specific problem of land cover classification. By using pre-trained models with large datasets and adapting them to a smaller and more specific one, the potential and generalization capability of the network to be adapted to another problem is exploited, with the advantage of using a reduced amount of training data and a significant improvement in computing time and computational cost (Gupta et al., 2022; Iman et al., 2023).

### Objectives

The primary objective of this study is to evaluate the potential of systematically applying data augmentation techniques within a deep learning (DL) framework for ultra-high-resolution (25 cm) land cover mapping. Given the significant challenges associated with manually annotating such data—an effort that is both time-consuming and resource-intensive—this study aims to identify the most effective augmentation strategies for improving model performance, especially when working with extremely limited training data. The study is unique in rigorously testing and ranking various augmentation techniques and combinations, offering a crucial framework for future researchers seeking to optimize model accuracy while minimizing manual labelling efforts.

The classification will be performed for three different years (2014, 2017, and 2020) over a 656 km^2^ area in the north of the Iberian Peninsula. The selection of training data will be deliberately constrained to a small fraction of the study area, testing the capacity of pre-trained models to generalize from minimal datasets—a scenario that mirrors the common challenge of working with limited labelled data in remote sensing.

To achieve this, eight distinct data augmentation techniques will be applied to generate a variety of training datasets. Each dataset will be used to train multiple models based on different neural network architectures. The study will produce a comprehensive ranking of these models, marking the first systematic comparison of data augmentation techniques in the field of land cover classification with very high-resolution imagery. The top-performing model from this analysis will then be used to generate land cover maps for the three target years. The accuracy and reliability of these maps will be validated against an independent dataset, ensuring the robustness of the findings and their applicability to other research scenarios.

## Materials and methods

### Study area and legend

The study area is located in the autonomous community of Cantabria, in the north of Spain. This region spans approximately 5321 square kilometres and is situated between the Cantabrian Sea and the Cantabrian Mountains. The study area covers 656 square kilometres in the central part of the region, and is characterized as a predominantly mountainous area covered mainly by vegetation, over which land management activities such as extensive livestock farming or forestry management have transformed the landscape.

The study area includes a part of the Saja-Besaya Natural Park. The vegetation in the area is dominated by lush forests of species such as oak, beech, and fir, as well as commercial species like eucalyptus or pine. The wooded areas are located in mid or high-mountain areas, followed by pastures interspersed with shrubland down to the valley floor, where the most productive grasslands prevail. The composition of the landscape in the chosen study area possesses great heterogeneity in both land cover and plant species, which will test the generalization capability of the classification algorithms and the augmentation techniques.

The combination of climatic and socioeconomic factors, such as rural depopulation, creates an environment conducive to vegetation succession, and gradually transforming grassland areas into shrublands. The spread of shrubland implies an increase in the accumulated fuel and the risk of fire, which also endangers the biodiversity of the territory and plant formations of high ecological value. To reduce the loss of pastures, practices of uncontrolled burning on shrubland areas are being carried out to recover grassland areas.

For this reason, high-resolution cartography is particularly relevant for monitoring such processes, as the transition from grass to shrubland is gradual and has a high degree of mixture that prevents medium-resolution sensors from detecting this process with sufficient anticipation and accuracy to mitigate it.

**Fig. 1:**
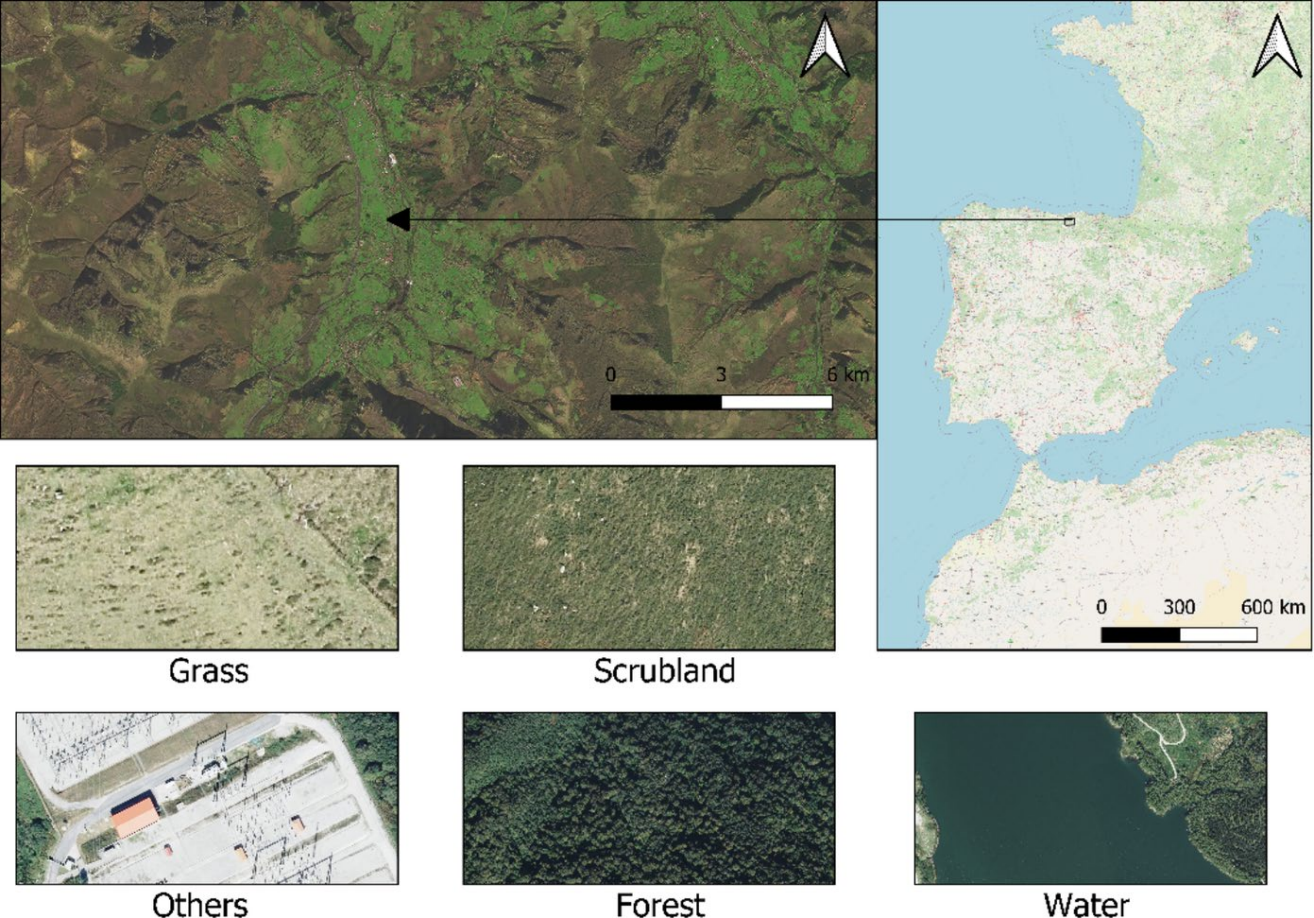
Detailed overview of the study area and the five analysis classes: Grass, Scrubland, Others, Forest, and Water

The legend for the land cover cartography of this work (Fig. 1) was selected considering the primary objective of this study, which is to evaluate and optimize data augmentation techniques to improve classification model accuracy. By maintaining a limited and manageable set of land cover classes, we ensure a rigorous assessment of the performance of different augmentation strategies without losing focus on the complexity of the classification task. This approach also aligns with similar medium-to-high-resolution products (Sentinel 2, 10–20 m) like ESRI’s Sentinel 2 product at 10 m or Google’s Dynamic World (DW) (Venter et al., 2022).

1. Grass: Pasture formations that develop both in the valleys and in the mountainous areas. These areas are typically occupied by livestock but are also used for fodder production.
2. Shrubland: Areas primarily covered by Gorse (Ulex sp.) and Heather (Erica sp.) with a height ranging from 50 cm to 2 m. These formations typically develop on abandoned pastures or transition areas and are prone to wildfires.
3. Forest: Areas predominantly occupied by tree species such as oak, beech, pine, eucalyptus, or fir. This class will include any isolated individual (tree).
4. Others: Represents all those elements that do not fit into the categories of pasture, shrubland, woodland and water. This allows for a clear visual representation of additional elements present in the study area, such as built structures, infrastructure, roads, shadows, or any other relevant non-natural element.
5. Water: Refers to water bodies, such as rivers, streams, small wetlands, or dam reservoirs, which are characteristics of the study area.

### High-resolution input data

The study area has very high-resolution images from the National Aerial Orthophotography Program (PNOA). These images provide a detailed representation of the land surface and are widely used in various applications, such as fire management (Montealegre et al., 2017), agricultural area analysis (Tomé Morán et al., 2013), cadastral mapping (Cuenca et al., 2016), or as base cartography. The PNOA provides orthomosaics of the entire country every 3 years. The PNOA images are characterized by having a spatial resolution of 25 cm with an XYZ precision ≤ 30 cm. They are distributed in raster format, combining red, green, and blue bands, and are encoded with a depth of 8 bits per band (RGB).

### Workflow

**Fig. 2:**
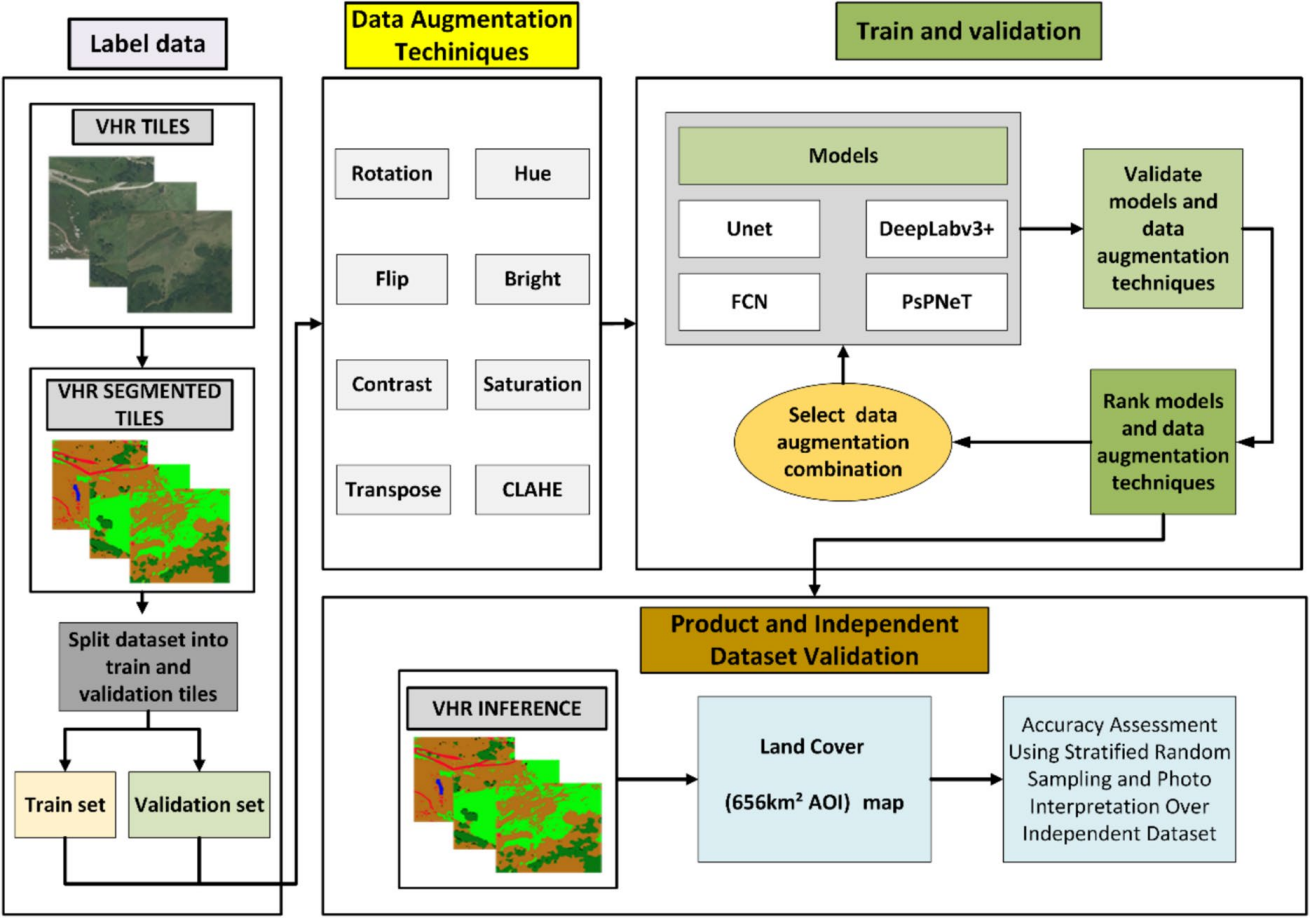
Workflow of the data augmentation and model training and validation process

Figure 2 shows the steps followed in the research process, from data labelling to result evaluation. The workflow commences with the acquisition of Very High Resolution (VHR) imagery, which is subsequently segmented into distinct categories and partitioned into training and validation datasets. These images are subjected to a range of data augmentation techniques, applied either singularly, in pairs, in triplets, or comprehensively. The augmentation techniques include rotation, transposition, flipping, brightness adjustment, contrast modification, saturation adjustment, hue alteration, and Contrast Limited Adaptive Histogram Equalization (CLAHE). The augmented datasets are then employed to train various image segmentation models, such as U-Net, DeepLabv3 + , Fully Convolutional Networks (FCN), and Pyramid Scene Parsing Network (PsPNet). Following this, the models and their respective augmentation techniques are validated and ranked based on performance metrics. The optimal combination of model and augmentation techniques is identified and subsequently tested in a designated study area. This involves performing inferences on the VHR images to produce high-resolution land cover maps. Finally, the accuracy of the generated cartography is validated to ensure the integrity and reliability of the mapping process, thus completing the workflow.

### Generation of training and testing data

The selection of data for calibration and validation involved a meticulous process of photo interpretation, encompassing an exhaustive review of the PNOA images throughout the study area. In this procedure, 26 regions that were representative of the territory’s heterogeneity and included all the classes in the legend were identified and selected. Each of these areas covers 0.05 km^2^, constituting 0.198% of the total extension of the study area.

The proportion of training data with respect to the territory’s extension was deliberately small to evaluate the generalization capability of the classification algorithms and analyze how data augmentation techniques can improve their results (Fujisawa et al., 2019; Ng et al., 2015). This decision is based on the premise that generating training data is a laborious and costly task. To optimize computational efficiency and ensure effective processing by the neural networks, a region size of 896 × 896 pixels was chosen. This size was selected based on the memory and processing limitations of the hardware used, ensuring that the available computational resources were not exceeded while still enabling efficient data handling. A semi-automatic approach was adopted to label the 26 selected areas. Initially, automatic segmentation of the images was carried out using a conventional algorithm, the multiresolution segmentation in QGIS with Orfeo Toolbox, grouping pixels based on their homogeneity using the RGB values of the PNOA images as input information. Subsequently, from these segments, a sample was selected to train a supervised classifier, specifically the nearest neighbour method. The result of this classification generated a preliminary map labelled with the classes in the legend. Later, photo interpretation was used to correct classification errors generated in the previous step.

This process was repeated for each area and year, resulting in a total of 78 labelled images. To train the model, 21 of the 26 areas (~ 80%) were chosen, leaving the remaining areas for validation (~ 20%) of the models. Since the classes of pasture, shrubland, and woodland are present in almost all 26 areas, classes like others and especially water are less common; the selection of areas for the validation and training set was carried out through random stratified sampling. This ensures that all classes are represented in both the validation and training set.

For testing the semantic segmentation model, the entire study area was selected, and a stratified sampling approach was implemented, encompassing 580 points distributed across the area over three different years. This method ensures a robust evaluation of the model’s performance by adequately representing spatial and temporal variations. Each of the pre-trained and validated segmentation models was tested using these sampling points. The results obtained from the model’s predictions were compared against the reference data at these points, enabling the assessment of accuracy, consistency, and the model’s ability to generalize across different temporal and spatial conditions within the study area. This process ensures that the selected model is not only accurate but also reliable and applicable across various epochs and regions within the area of interest.

### Data augmentation techniques

The application of data augmentation techniques has emerged as an essential component in the field of deep learning applied to land cover mapping, as it enhances the efficacy of machine learning models (Imbert, 2019; Yuan et al., 2021). Mapping land cover through high-resolution images presents significant challenges due to the limited availability of labelled data and the inherent variability of the images captured at different times under different acquisition conditions (e.g. lighting, sun position, shadows (Stivaktakis et al., 2019)). In this context, data augmentation acts as a strategy to mitigate these limitations, allowing for the generation of more diversified and representative training sets.

In this study, two typologies of data augmentation techniques were applied: radiometric and geometric. Radiometric techniques are designed to address potential variations in colour that a surface might experience due to factors such as phenology or lighting conditions at the time of image capture. On the other hand, geometric techniques seek to vary the perspective from which the surface is observed. These geometric transformations significantly complement the visual characteristics of the original image. Each of the datasets will contain the original images and the images generated using the techniques or combination of techniques described. Specifically, the performance of the following data augmentation techniques will be evaluated:

- Rotation: This technique involves rotating the image by a certain angle. It can help create variations in the orientation of objects present in the image. A random angle of rotation is applied to the image between − 90 and + 90°.
- Transposition: This involves swapping the rows for columns in the image, which can generate subtle changes in the appearance of the image.
- Flipping: This technique involves flipping the image horizontally or vertically. It can help simulate different perspectives and orientations.
- Contrast: Adjusting the contrast involves changing the difference between the brightness values of the pixels in an image. It can make objects stand out more or less depending on the setting. It can soften the image and reduce details, simulating conditions of diffuse or cloudy lighting. A random percentage is applied to the base contrast of the image between − 20 and + 20%.
- Brightness: Changing the brightness of an image involves globally adjusting the level of lighting. It can make the image lighter or darker as a whole, which can simulate conditions of intense lighting or sunsets. A random percentage is applied to the base brightness of the image between − 15 and + 15%.
- Saturation: Adjusting the saturation involves changing the intensity of the colours in an image. It can make the colours more vibrant or muted. It can simulate conditions of intense lighting. A random percentage is applied to the base saturation of the image between − 20 and + 20%.
- Hue: Changing the hue of an image involves adjusting the colours on the colour wheel. It can generate variations in the appearance of objects by changing the predominant colours. A random angle of hue is applied to the image between − 10 and + 10 degrees.
- CLAHE (Contrast Limited Adaptive Histogram Equalization): This technique involves enhancing local contrast in an image by applying histogram equalization in small regions rather than the entire image at once. It helps to highlight details in areas of different lighting levels.

**Fig. 3:**
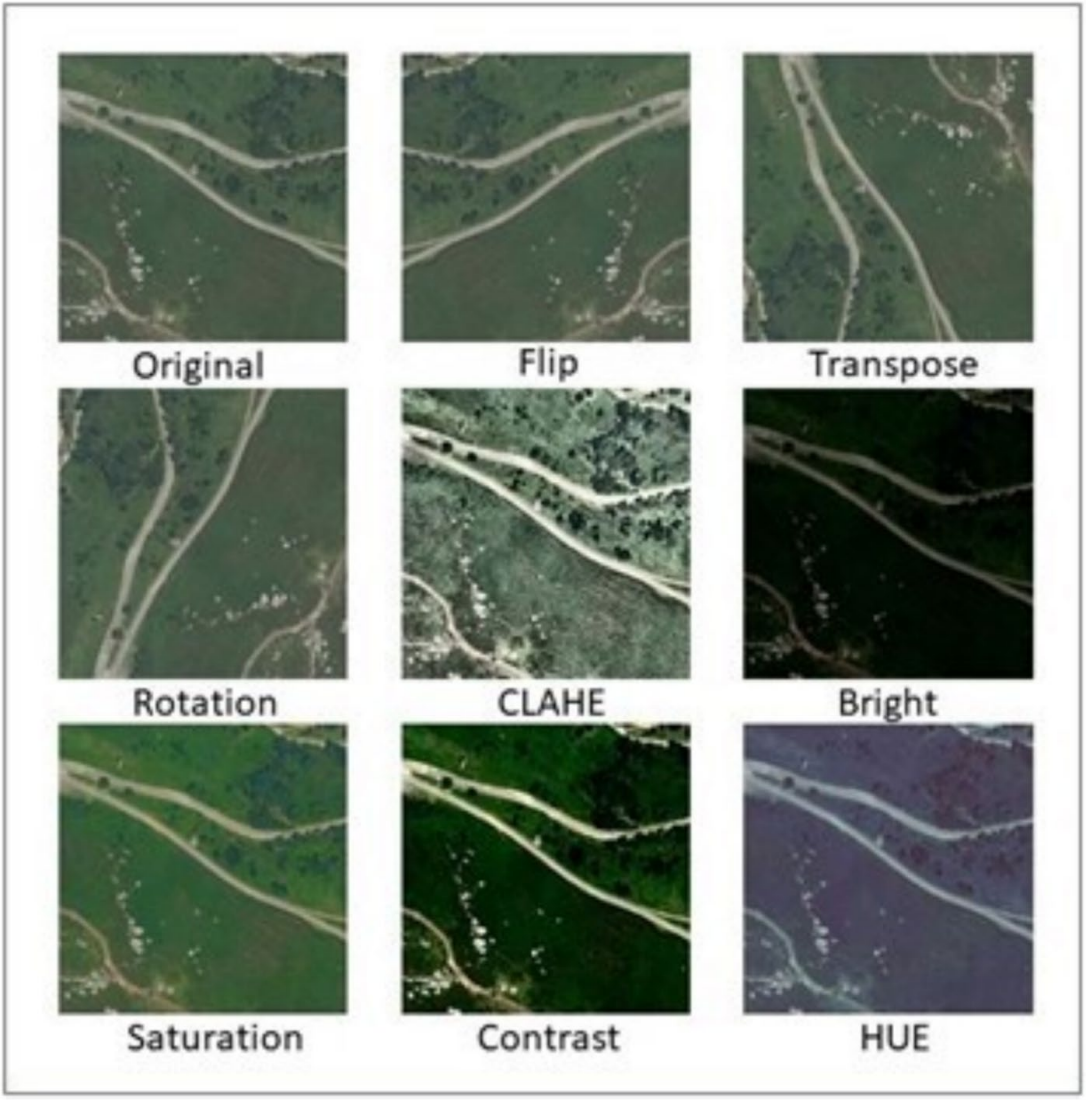
Original image and eight data augmentation techniques: Flip, Transpose, Rotation, CLAHE, Brightness, Saturation, Contrast, and Hue Adjustments

Figure 3 shows the changes applied to the original image by each of the data augmentation techniques used during the study. These techniques can be applied individually to the dataset, doubling the amount of training data with each method applied, but they can also be combined by applying several modifications at the same time to obtain a completely different image. In this article, with the intention of testing the individual power of each technique, we began by applying only one technique to each image at the same time. This way, the dataset to which a single data augmentation technique is applied will have twice as many images (126) as the original dataset (63), the dataset with two techniques will contain three times (189) as many as the original dataset, the dataset with three techniques will have four times more images (252), and finally, the dataset with all techniques contains ten times more images than the original dataset (630). To assess their performance, a model will be trained and validated for each new training dataset generated. The goal is to determine which method or combination of methods of data augmentation works best for a specific network architecture.

Each of the techniques was tested and compared separately (radiometry and geometry), the combination of two techniques, joining the best of radiometry and geometry, three techniques, joining the best combinations of two techniques with the techniques that appear most often in the top ranking of combinations using all techniques together in a single dataset. To simplify the analysis, the sets of more than one technique were combined by joining the best radiometric and geometric techniques for the ranking of each model. The last combination involves generating a dataset that contains all the individual techniques, both radiometric and geometric.

### Selection of network architectures

Four different semantic segmentation models have been selected for applying the various training sets generated according to the section “Data augmentation technique”. All models are included in the MMSegmentation platform (Mms Contributors, 2020), an open-source library developed by the Megvii Research team, which provides a wide range of state-of-the-art models and algorithms for semantic segmentation in images. It is based on the DL framework PyTorch and offers an efficient and flexible implementation of popular architectures such as U-NET, DeepLabV3 + , PSPNet, and FCN, among others. One of the main strengths of MMSegmentation lies in its ability to test and explore different segmentation models and adjust hyperparameters, such as learning rate, batch size, and loss function. The models were selected due to their potential in different tasks, their generalization capability, and their ability to achieve good results with few data (Chen et al., 2018; Zhang et al., 2021; Zhao et al., 2017). Transfer learning was employed for each of the models to leverage pre-trained ResNet50 weights, which helps enhance performance, particularly given the limited dataset, and ensures consistency across the study. The selected models, summarized in Table 1, are the following:

- U-NET (Ronneberger et al., 2015) is a neural network architecture for semantic segmentation consisting of an encoder and a decoder. The encoder uses convolutional layers to extract features and reduce spatial resolution, while the decoder uses transposed convolutional layers to increase resolution and generate a detailed output. The shape of U-NET resembles an inverted “U” with direct connections between the encoder and decoder to preserve contextual information. It is efficient in terms of memory and stands out in medical applications (Ronneberger et al., 2015) and satellite image recognition (Ch et al., 2022).
- DeepLabV3 + (Chen et al., 2018) is an architecture for semantic segmentation that uses an encoder-decoder structure with convolutional layers. The encoder extracts features using a network such as ResNet101, while the decoder uses transposed convolutions and atrous to increase resolution and generate a detailed output. DeepLabV3 + employs Atrous Spatial Pyramid Pooling (ASPP) to capture features of different sizes and a class balance technique to address imbalances in the training set. It is known for its accuracy and is used in a variety of computer vision applications applied to remote sensing (Du et al., 2021; Wang et al., 2022).
- FCN (Fully Convolutional Network)) (Long et al., 2015) is a neural network architecture designed for semantic segmentation. It uses convolutional and pooling layers to extract features and produces an output that maintains spatial resolution. Instead of using fully connected layers, FCN uses transposed convolutions to increase resolution and generate a detailed segmentation mask. In this case, ResNeSt101, the most modern of the feature extraction networks in the study, is used. FCN is versatile and has been applied in various domains, such as autonomous driving and aerial or satellite image segmentation (Li et al., 2021; Xia et al., 2021).
- PSPNet (Pyramid Scene Parsing Network) (Zhao et al., 2017) is a neural network architecture for semantic segmentation that uses a pyramid structure to capture contextual information at different scales. It consists of a backbone (such as ResNet-101), a Pyramid Pooling Module (PPM), and a decoder. The PPM aggregates features from multiple scales, allowing the network to process contextual information at different levels of granularity. The decoder uses this aggregated feature information to generate the segmentation mask. PSPNet is known for its ability to capture multi-scale context and is widely used in high-level segmentation tasks (Li et al., 2021; Xia et al., 2021).

**Table 1:** Comparison of semantic segmentation models: architectures, strengths, and limitations

Model	Architecture	Advantages	Disadvantages	Trainable parameters and applications
**DeepLabV3 + **	Encoder-decoder architecture with atrous convolution and spatial pyramid pooling	High performance for large-scale datasets	Computationally expensive	~ 42 million
		Good handling of object boundaries	Requires large memory	Autonomous driving
		Effective in multi-scale feature extraction using atrous convolutions	Struggles with small objects in complex scenes	Medical image segmentation
**FCN**	Fully convolutional network, typically with VGG or ResNet backbone	End-to-end learning	Struggles with accurate boundary detection	~ 134 million
		Effective for large input images due to its fully convolutional nature	Lack of global context, especially in complex scenes	Satellite image segmentation
**U-Net**	Symmetric encoder-decoder architecture with skip connections	Good for small datasets	Struggles with complex textures and large-scale images	~ 31 million
		High accuracy in medical image segmentation	Overfitting on small datasets	Biological image analysis
**PSPNet**	Pyramid pooling network with multi-scale context aggregation	Strong multi-scale context capture	High computational cost	~ 61 million
		Handles large variations in scale	Requires large datasets for training	Autonomous driving

### Model scalability, transfer learning, and computational considerations

Our framework is designed to be fully scalable, which makes it highly adaptable to various contexts and datasets. Similar architectures have been successfully applied to datasets with different resolutions in numerous studies, achieving promising results. This scalability is particularly important for addressing a wide range of land cover types and resolutions, allowing the framework to be applied across diverse geographical areas. Studies such as Neupane et al. (2021) work with images at a resolution of 5 cm, while Garioud et al. (2022) utilize images at 20 cm resolution. In our study, we employ images at 25 cm resolution. Additionally, Du et al. (2021) work with multispectral images that include RGB bands, but at resolutions greater than 1 m. This suggests that the lower the resolution, the more data is needed for classification, especially when distinguishing between classes with subtle differences. This highlights the scalability and adaptability of our framework, which can effectively handle datasets of varying resolutions, from high to low, by incorporating more comprehensive data for challenging classification tasks. In our study, we utilized **transfer learning** by leveraging a **ResNet-50** model pre-trained on **ImageNet**. This approach enabled us to fine-tune the model specifically for our task, capitalizing on the feature extraction capabilities of the pre-trained model. By adapting the model to the characteristics of our dataset, we were able to accelerate the learning process and improve performance, particularly given the relatively small size of our dataset. Regarding the applicability of the model in other regions, we believe that it can perform well in areas with land cover similar to our study region (e.g. landscapes resembling Cantabria, such as parts of northern Spain, southern France, or western Portugal). With minimal adjustments, the model could be reproduced and applied to other regions with comparable environmental conditions. However, it is important to note that the primary objective of our study is not to create a universally applicable model but to demonstrate that accurately labelling a small dataset—specific to any given region—can yield highly reliable results. This emphasizes the potential of our approach to achieve strong local performance as long as sufficient representative data from the target area is available for fine-tuning.

Despite its scalability and flexibility, the computational requirements for implementing this framework are significant. The techniques used in our study are most suitable for smaller-scale areas, such as provinces or cities, rather than large national or continental regions. For example, training our model on a **NVIDIA GeForce RTX 2080 GPU with 8 GB of VRAM** took approximately **3 h**. But once the model was trained, inferring the **AOI of 656 km**^**2**^, comprising **13,000 images**, took only an estimated **16.25 min**, with an average processing time of **75 ms per image**. These computational considerations are critical when applying the framework to larger areas and highlight the importance of having efficient computational resources for scaling the model.

### Testing of the best models and techniques and final model selection

In this phase, the objective is to develop a ranking system to evaluate the performance of various neural network architectures when combined with different data augmentation techniques. The aim is not only to identify the most effective architecture for handling small-sized datasets in land cover mapping but also to determine the most efficient data augmentation methods. This will help streamline future research efforts, saving time for other investigators working on similar tasks.

For each combination of neural network and data augmentation technique, a model will be trained, and its performance will be evaluated using the same validation dataset. Two key metrics will be used for this evaluation: **accuracy** and the **Intersection over Union (IoU)**, also known as the Jaccard Index.

1 $${\text{IOU}}=\frac{\text{Intersection area}}{\text{Union area}}$$

2 $${\text{Accuracy}}=\frac{\text{Number of correct predictions}}{\text{Number of predictions}}$$

While both metrics are important, IoU is preferred as the primary indicator in this study because it provides a more sensitive measure for pixel-level segmentation and object delineation errors, which is crucial in land cover mapping. **IoU** is especially valuable when precise object localization is critical, making it more representative than overall accuracy for this specific task.

To assess the impact of data augmentation, we also trained the models using the original dataset (without augmentation) and calculated the percentage of improvement in model performance when augmentation was applied. In addition to the general improvement in overall precision and IoU, we conducted an independent analysis of these metrics, calculating their respective percentages of improvement to provide a more detailed understanding of the augmentation's contribution.

3 $$\mathrm{Improvement}\;\left(\%\right)=100\left(\left(\frac{{\mathrm{Acc}}_{\mathrm{Aug}}}{{\mathrm{Acc}}_{\mathrm{Base}}}+\frac{{\mathrm{IoU}}_{\mathrm{Aug}}}{{\mathrm{IoU}}_{\mathrm{Base}}}\right)/2-1\right)$$

4 $$\text{Improvement Accuracy}\left(\text{\%}\right)=100\left(\frac{{\text{Acc}}_{\text{Aug}}}{{\text{Acc}}_{\text{Base}}}-1\right)$$

5 $$\text{Improvement IoU }\left(\text{\%}\right)=100\left(\frac{{\text{Iou}}_{\text{Aug}}}{{\text{Iou}}_{\text{Base}}}-1\right)$$

where:

Acc_Aug_ is the accuracy achieved by the model evaluated with data augmentation techniques.

Acc_Base_ is the accuracy achieved by the base model.

IoU_Aug_ is the IoU achieved by the model evaluated with data augmentation techniques.

IoU_Base_ is the IoU achieved by the base model.

To ensure a fair comparison between models and augmentation techniques, all models were trained under the same conditions: 500 iterations, with a batch size of 16 images per iteration, resulting in a total of 8000 images processed by each model during training. It is important to clarify that the total of 8000 images refers to the number of images processed throughout the training process, regardless of whether data augmentation was applied. When data augmentation is used, the diversity of the training data increases as the techniques generate variations of the original images. This added diversity helps delay overfitting, which tends to occur more quickly when training on smaller datasets where the same images are repeated frequently. The choice to use 500 iterations was made because the results obtained with this number were satisfactory. Training each model until early stopping would have been too demanding in terms of time and computational resources, given that the study involved training a total of 72 models (18 different datasets and 4 models). Furthermore, using early stopping would have introduced disparities in the number of images used to train each model, compromising equality in the comparison of results. We selected this fixed number of iterations because preliminary experiments showed that overfitting in the original dataset (without augmentation) began to occur near this threshold. By setting this limit, we ensured a consistent evaluation while avoiding overfitting in all cases. This methodology provides a reliable basis for comparing the performance of different architectures and augmentation methods.

### Selection of the best model and subsequent evaluation in cartography generation

Once the ranking was completed, the model with the highest IoU was selected, as this is the most representative metric for segmentation tasks. As previously mentioned, efforts were made to equalize conditions as much as possible for evaluating the models and applying data augmentation techniques. To ensure a fair comparison, all models were trained for a fixed number of 500 iterations, regardless of their augmentation techniques or dataset size.

While this approach ensures consistency across models, it also means that the optimization of the best-performing model—trained with the highest diversity of data through the combination of augmentation techniques—may not have reached its full potential within the 500 iterations. Given the richer dataset, this model likely requires more iterations to fully capitalize on the additional information and reach its maximum performance.

In this final evaluation phase, the model’s performance was assessed using accuracy and IoU metrics, along with the creation of a confusion matrix to ensure a comprehensive analysis.

#### Generation of land cover cartography

Generating LC maps from very high-resolution satellite images requires intensive processing with a high computational cost. Furthermore, as these are extremely large images, the network resamples when ingesting the data, potentially losing relevant information. For this reason, the tiling of images was performed at 896 × 896px. Tiling the study area into smaller images addresses the potential computational limitations of the equipment or the model.

During the individual segmentation of each tile, each one is processed independently, considering only its local context. This approach carries the possibility of border zones being classified using different criteria, which could result in inconsistent classifications at the border edges between tiles or when generating a mosaic with all of them. To avoid this issue, the study area was subdivided with 50% overlaps, that is, overlapping areas of 448 × 448 pixels. This overlap allows each tile to share a significant context with its neighbour, thus reducing the impact of the edge effect on the generation of the final mosaic.

#### Testing land cover cartography

**Fig. 4:**
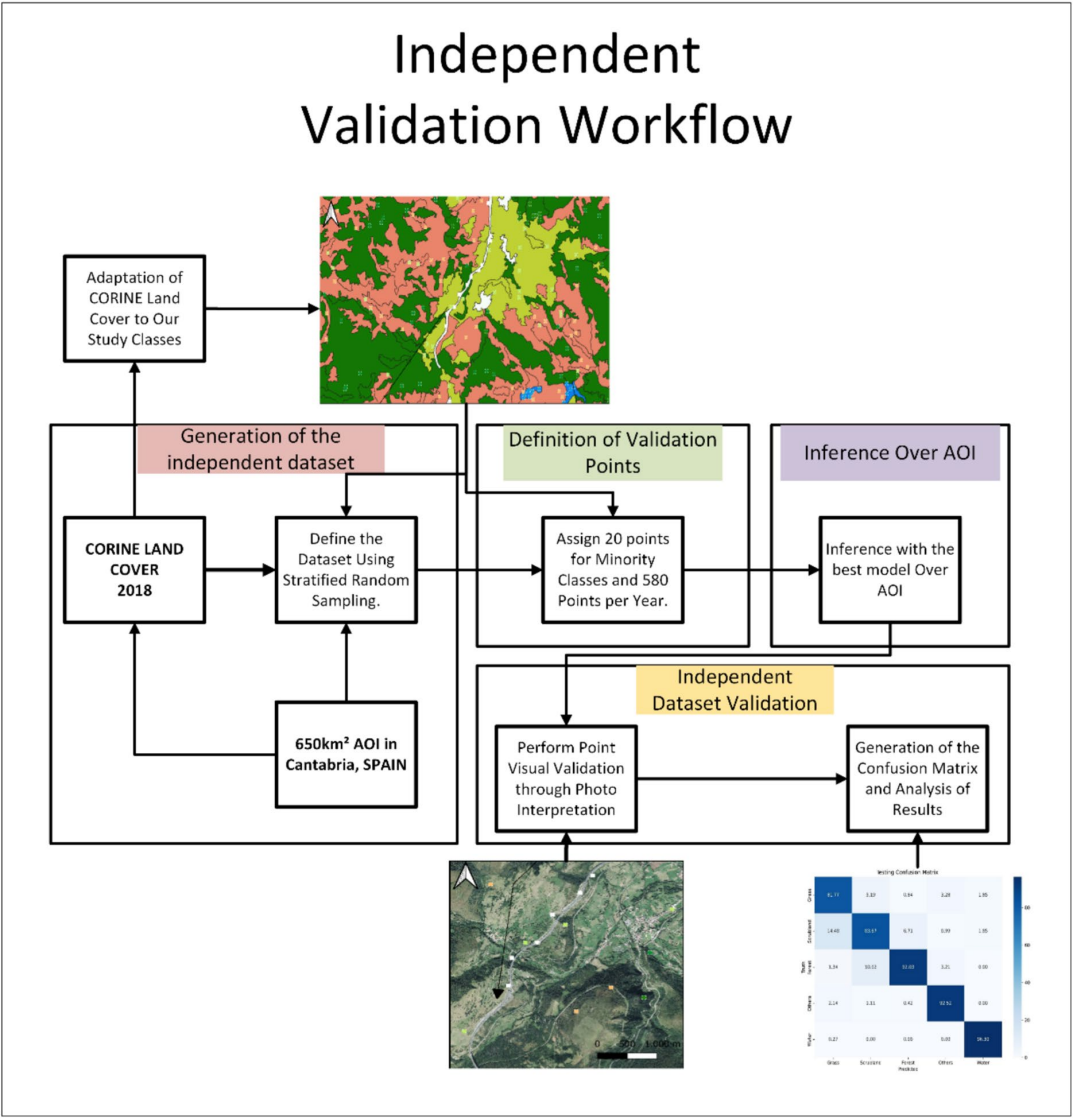
Independent dataset validation workflow

The accuracy of the model outputs was assessed using an independent dataset that was not involved in the training process. This independent dataset was created through a stratified random sampling approach, as outlined in the workflow presented in Fig. 4. The sampling units were output pixels, and the strata were defined based on the land cover classes from the CORINE Land Cover map of 2018 (EEA, 2019), which were adapted to align with the land cover classes used in our study. This adaptation ensured consistency between the CORINE classes and the specific classes analyzed in this research. The stratification ensured that all classes were adequately represented in the testing dataset. For minority strata, such as water, a minimum of 20 points per year were designated to ensure these classes were well represented in the validation. This approach significantly increased the testing area, as the validation set is sparser, thereby improving the overall validation process. The testing was conducted over the entire area of interest (AOI), covering approximately 656 km^2^, ensuring comprehensive evaluation across the study region. The accuracy assessment involved photo interpretation of high-resolution images to validate the model’s predictions. A total of 580 pixels per year were analyzed, resulting in 1740 validation points in total. The validation was carried out by extracting the actual value of the cartography at each point and comparing it with the value predicted by the model. A confusion matrix was generated to represent the omission and commission errors for each class.

This dataset is distinct from the 20% of labelled data used for model validation during training, as described in Section 2.4. While the latter was part of the training process to monitor and refine the model, the independent dataset described here was exclusively reserved for evaluating the final performance of the model outputs.

## Results

The resulting datasets from the combinations can be seen in Table 2; these datasets were evaluated against a common validation set.

**Table 2:** Best combinations of data augmentation techniques

Base dataset	One technique	Two techniques	Three techniques	All together
Original dataset	Rotation	Rotation + contrast	Flip + contrast + bright	
	Transpose	Flip + contrast	Flip + bright + CLAHE	
	Flip	Flip + bright	Rot + contrast + CLAHE	
	Contrast	Flip + CLAHE		
	Bright	Rotation + CLAHE		
	Saturation			
	Hue			
	CLAHE			

### Ranking of models and datasets

Table 3 shows the baseline results from training the four deep learning models (without data augmentation). Although DeepLabV3 + demonstrated superior performance in maintaining the shape of the segments, U-NET achieved a slightly better overall accuracy.

**Table 3:** Metrics of best training with base dataset

Model	Accuracy	IoU
DeepLabV3 +	0.723	0.495
U-NET	0.741	0.48
FCN	0.737	0.474
PSPNet	0.728	0.452

The separate analysis of both radiometric and geometric data augmentation techniques reveals a clear hierarchy in their ability to enhance the performance of image segmentation models. Among the techniques evaluated, **rotation**, **flip**, **brightness adjustment**, **contrast**, and **CLAHE** consistently stand out as the most effective, achieving significant improvements in accuracy and Intersection over Union (IoU). This consistency across different architectures suggests that these techniques not only optimize specific aspects of the models but also enhance the overall ability of the networks to capture spatial and radiometric features in the images. It is important to note that the **PSPNet model** was excluded from the study, as no technique demonstrated a real improvement in its results.

Table 4 presents the performance metrics for each model using a single data augmentation technique. **Rotation** proved to be the most effective technique for **U-Net**, while **contrast variation** worked best for **DeepLabV3 + **. Based on these results, two techniques **HUE** and **Saturation** were also discarded, as they showed negative impacts on model performance.

**Table 4:** Metrics obtained for the best trainings with a single data augmentation technique

Model	Technique	Accuracy	IoU	% improvement	%Acc improvement	%IoU improvement
U-NET	Rotation	0.779	0.623	17.46	5.13	29.79
U-NET	Bright	0.748	0.596	12.56	0.94	24.17
U-NET	Flip	0.755	0.553	9.55	1.89	15.21
U-NET	HUE	0.71	0.44	− 6.26	− 4.18	− 8.33
DeepLabV3 +	Contrast	0.784	0.598	14.62	8.44	20.81
DeepLabV3 +	Flip	0.788	0.597	14.8	8.99	20.61
DeepLabV3 +	Saturation	0.709	0.45	− 5.51	− 1.94	− 9.09
FCN	CLAHE	0.742	0.57	10.47	0.68	20.25
FCN	Rotation	0.764	0.567	11.64	3.66	19.62
FCN	Bright	0.755	0.553	9.55	2.44	16.67
PSPNet	Transpose	0.745	0.394	− 5.23	2.34	− 12.83
PSPNet	Flip	0.676	0.373	− 12.34	− 7.14	− 17.48
PSPNet	Contrast	0.703	0.367	− 11.09	− 3.43	− 18.81

The results of training each model with two combined data augmentation techniques, as shown in Table 5, demonstrate even more substantial improvements compared to the single technique experiments. The U-Net model showed outstanding performance with the rotation + contrast technique, achieving an overall improvement of 28.14% and a 43.33% improvement in IoU, making this combination the most effective augmentation. Notable improvements were also observed with rotation + brightness, which presented a 27.69% overall improvement and a 42.71% improvement in IoU. The combinations of rotation + CLAHE and flip + contrast showed more moderate improvements, with increases ranging from 22.16 to 24.59% in overall improvement and improvements of 35.42 to 38.13% in IoU. These techniques highlight that the U-Net model responds positively to augmentations that enhance image features, particularly those involving rotation and contrast. In the case of Deeplabv3 + , the flip + CLAHE combination stood out, achieving a 27.22% overall improvement and a 37.58% improvement in IoU. The model showed very similar performance with the combinations of rotation + CLAHE and flip + contrast, both with improvements of 27.22% and 37.58% in IoU. The rotation + brightness technique also produced good results with a 26.93% overall improvement and a 37.17% improvement in IoU. These results suggest that Deeplabv3 + is particularly sensitive to augmentations involving contrast enhancement, and that rotation and flip techniques are equally effective in this model. The FCN model presented the flip + contrast technique as the most effective, with a 31.75% improvement and 41.77% in IoU, showing a considerable improvement compared to the other augmentation combinations. The combinations of flip + CLAHE and flip + brightness were also effective, with a 26.52% improvement in accuracy and an increase of 39.87% to 41.77% in IoU. While the FCN model showed good results, especially with flip + contrast, its overall performance was slightly lower than that of Deeplabv3 + and U-Net, though still competitive in terms of accuracy and IoU.

**Table 5:** Results training each model using two data augmentation techniques

Model	Technique	Accuracy	IoU	% improvement	%Acc improvement	%IoU improvement
U-NET	Flip + contrast	0.837	0.688	28.14	12.96	43.33
U-NET	Flip + bright	0.835	0.685	27.69	12.69	42.71
U-NET	Rotation + CLAHE	0.823	0.663	24.59	11.07	38.13
U-NET	Rotation + contrast	0.814	0.66	22.16	8.91	35.42
U-NET	Flip + CLAHE	0.798	0.63	20.59	7.69	31.25
Deeplabv3 +	Flip + contrast	0.837	0.688	27.38	15.77	38.99
Deeplabv3 +	Flip + CLAHE	0.845	0.681	27.22	16.87	37.58
Deeplabv3 +	Rotation + CLAHE	0.845	0.681	27.22	16.87	37.58
Deeplabv3 +	Rotation + contrast	0.841	0.679	26.93	16.32	37.17
Deeplabv3 +	Flip + bright	0.841	0.673	26.14	16.32	35.96
FCN	Flip + contrast	0.836	0.672	31.75	13.43	41.77
FCN	Flip + CLAHE	0.834	0.663	26.52	13.16	39.87
FCN	Flip + bright	0.834	0.663	26.52	13.16	39.87
FCN	Rotation + CLAHE	0.829	0.657	25.55	12.48	38.61
FCN	Rotation + contrast	0.821	0.645	23.74	11.40	36.08

After analyzing the results of the two-technique combinations, combinations of three data augmentation techniques were selected based on their repeated appearances in the ranking and their consistent performance across different models.

Testing with three data augmentation techniques (Table 6) revealed that the best combination was flipping, contrast enhancement, and brightness. However, the improvements were not significantly larger compared to the combinations using just two techniques. This suggests that adding more techniques does not always lead to better performance. To confirm these results, we plan to test the efficacy of utilizing all augmentation techniques simultaneously. This will help us determine if there is a point at which additional techniques no longer contribute to model generalization.

**Table 6:** Training results of each model using three data augmentation techniques after 500 iterations

Model	Technique	Accuracy	IoU	% improvement	%Acc improvement	%IoU improvement
DeepLabV3 +	Flip + contrast + bright	0.837	0.69	27.58	15.77	39.39
DeepLabV3 +	Flip + contrast + CLAHE	0.83	0.659	23.97	14.80	33.13
DeepLabV3 +	Rot + contrast + bright	0.784	0.615	16.34	8.44	24.24
FCN	Flip + contrast + CLAHE	0.83	0.658	25.72	12.62	38.82
FCN	Flip + contrast + bright	0.827	0.658	25.52	12.21	38.82
FCN	Rot + contrast + bright	0.801	0.61	18.69	8.68	28.69
U-NET	Rot + contrast + CLAHE	0.813	0.65	22.57	9.72	35.42
U-NET	Flip + contrast + bright	0.772	0.598	14.38	4.18	24.58
U-NET	Flip + contrast + CLAHE	0.779	0.57	11.94	5.13	18.75

Finally, Table 7 shows the results when all augmentation techniques were applied simultaneously. Surprisingly, performance degraded significantly across all models, confirming that using too many augmentation techniques at once can lead to poorer results, likely due to increased noise or overfitting from learning unrealistic transformations of limited training data.

**Table 7:** Metrics obtained for the best training with all data augmentation techniques

Model	Technique	Accuracy	IoU	%Improvement
DeepLabV3 +	All together	0.744	0.615	13.57
U-NET	All together	0.732	0.592	11.05
FCN	All together	0.68	0.563	5.52
PSPNet	All together	0.65	0.385	− 12.76

Single data augmentation techniques improved model performance by up to 17.46% with rotation and contrast variation being the second with a 14.62% improvement, proving to be the most effective methods. When combining two techniques, the improvements were even more significant, reaching up to 31.75%. In particular, the combination of horizontal flipping, brightness adjustment, and contrast variation emerged as the most successful strategy. However, adding a third technique resulted in diminishing returns, and applying all techniques simultaneously led to a notable drop in performance. Overall, the DeepLabV3 + model, when trained with a combination of horizontal flip, contrast, and brightness augmentation, delivered the best results, making it the most promising model for generating land cover maps in this study.

At the beginning of the study, it was assumed that radiometric changes would not significantly affect model performance, as the images had undergone consistent radiometric corrections. However, it was observed that between 2014 and 2021, improvements in camera technology resulted in these corrections affecting the images differently over time. This highlights the importance of including data augmentation techniques that account for these variations during training, ensuring that the model remains functional despite evolving imaging technology. Such techniques not only improve the model’s ability to generalize across different acquisition periods but also ensure its robustness for future campaigns, which will likely face similar shifts due to continued advancements. This approach helps the model remain adaptable and effective over time, supporting its long-term functionality.

### Extended training of the selected model

After selecting the best-performing model, an extended retraining was conducted for 5000 iterations to maximize performance while avoiding overfitting by using early stopping. The hyperparameters for the retrained model are detailed in Table 8.

**Table 8:** Hyperparameters of the best model

Best iteration	3800
Best epoch	312
Data augmentation combination	Flip + contrast + bright
Batch size	16
Number of images	195
Number of iterations	5000
Training epochs	200
Learning rate	0.0001
Optimization algorithm	SGD
Input size	896 × 896 × 3
Classes	5
Depth	4
Filters on the first level	64
Padding	Yes
Backbone architecture	Resnet101

The results on the validation set showed a significant improvement in model performance while the initial 500 iterations provided a good approximation; they were insufficient to fully optimize the model. After 3800 iterations, the model achieved an accuracy of 0.89 and an IoU of 0.78 on the validation dataset, reflecting a 41% improvement compared to the base dataset. However, compared to the best model trained with 500 iterations, the performance gain was 5.3% for precision and 9% for IoU. This smaller but noticeable increase demonstrates that while 500 iterations give a solid benchmark, further training yields more refined results.

**Fig. 5:**
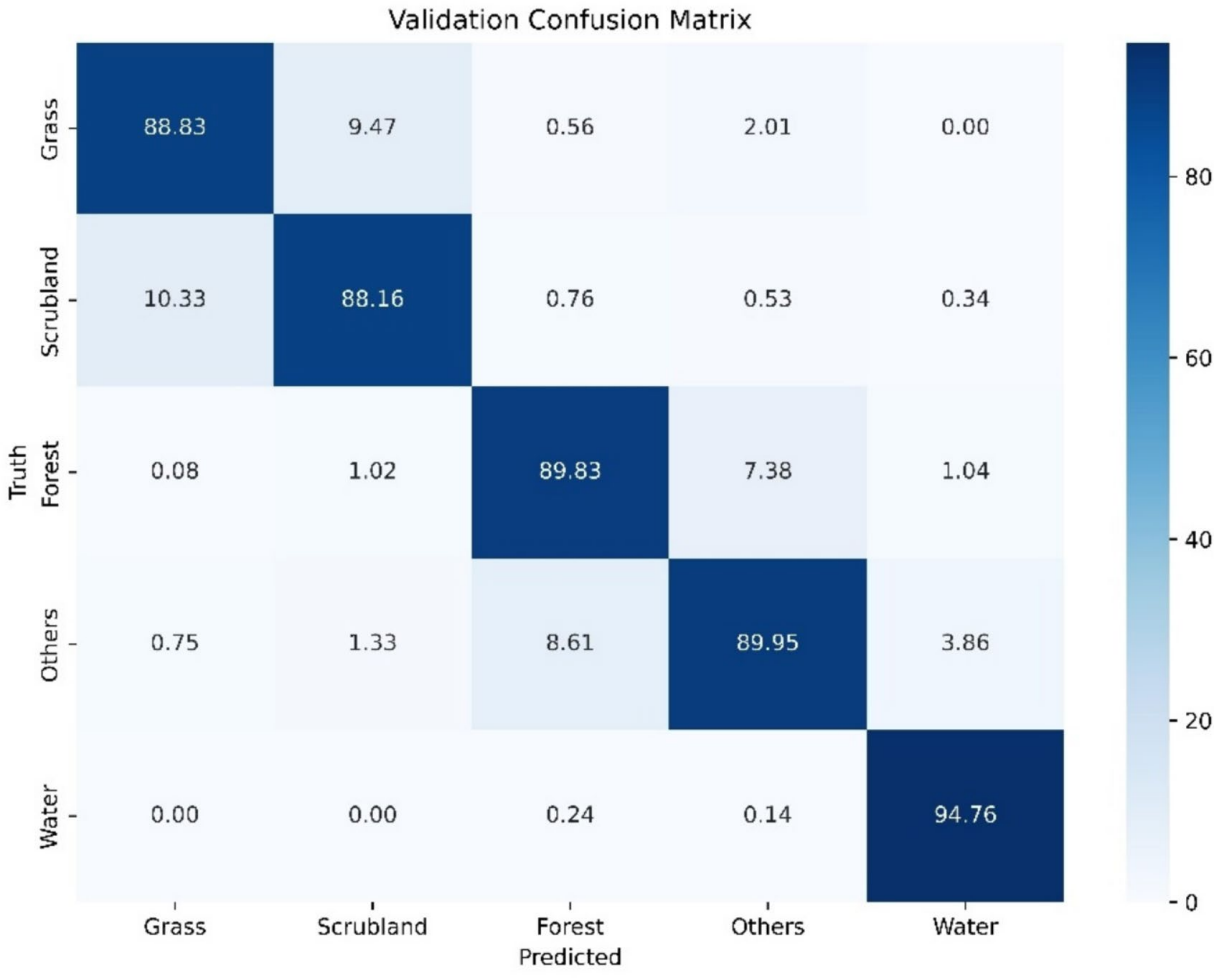
Validation results using the validation set and the exhaustively trained model

Figure 5 presents the confusion matrix generated during the validation phase. All classes demonstrate high accuracy, with most above 88%, and Water achieving the highest at 94.76%. However, the most confusion occurred between shrubland and pasture classes, which is likely due to their shared characteristics. Additionally, confusion between the Forest and Others categories is linked to the inclusion of shadows within the Others class, which are often present in forested regions.

**Fig. 6:**
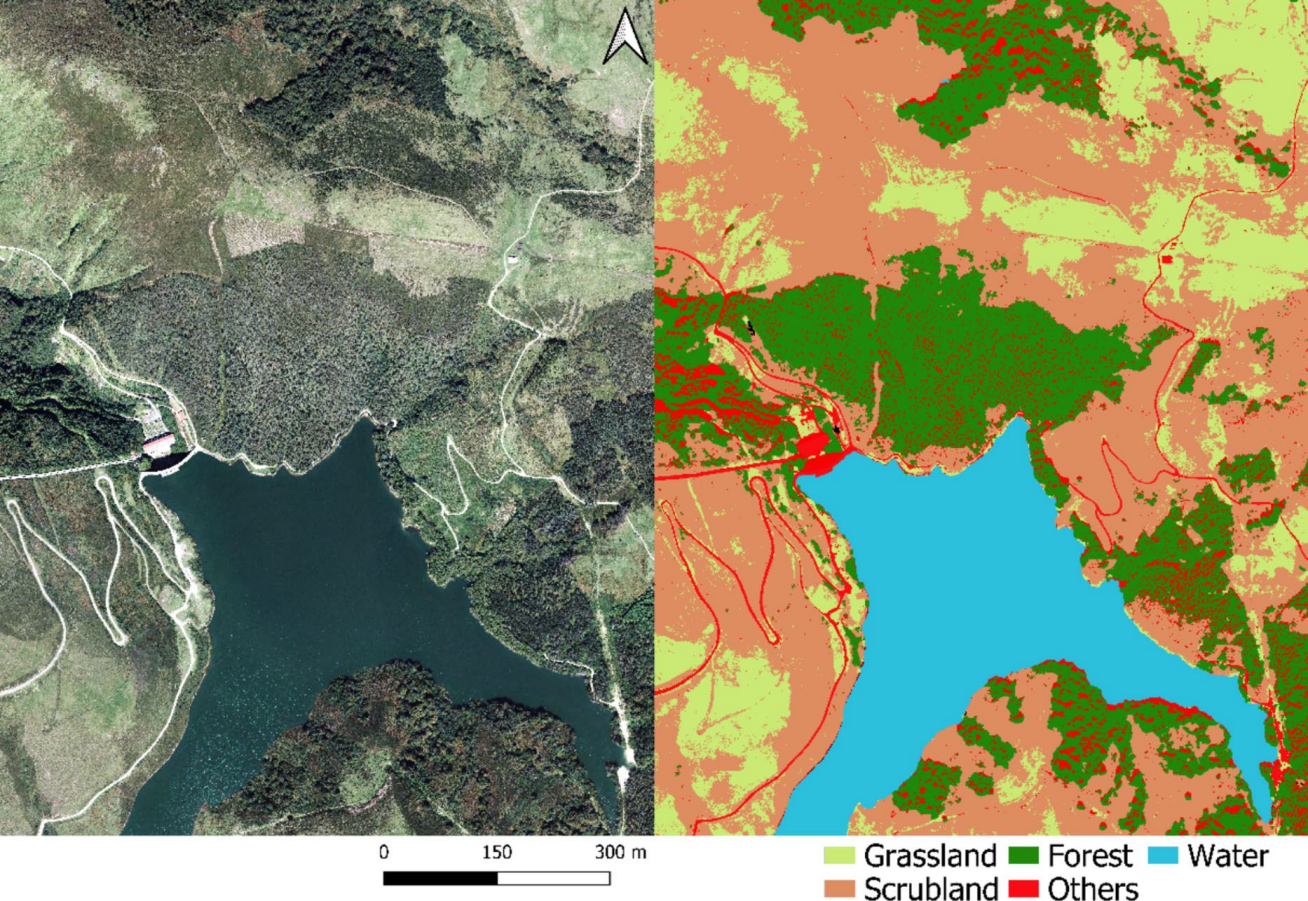
Example of cartographic inference: A region and the corresponding land cover inference

Figure 6 shows an example of the model’s inference on a portion of the study area. The model’s ability to classify different land cover types at a high level of detail is evident. During independent testing, conducted over 1740 pixels, the model’s metrics remained consistent with those from the training phase. Some variations in accuracy, particularly for pasture and shrubland, are attributed to the test set’s location near areas undergoing land cover changes. Since the validation process focused on pixel-level accuracy, areas near class borders were more prone to misclassification.

**Fig.7:**
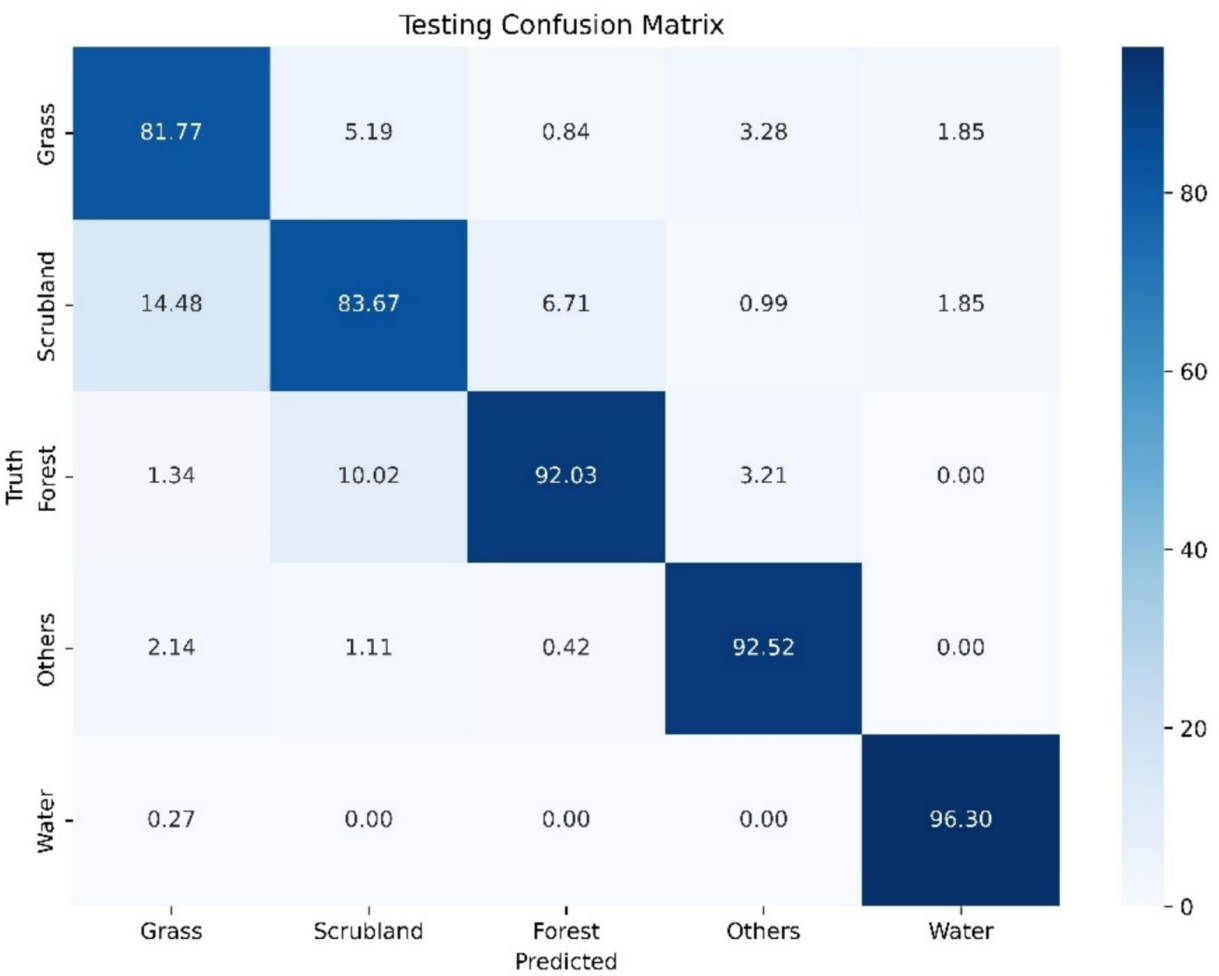
Confusion matrix derived from 1740 photo-interpreted points of CORINE Land Cover class stratification

The test confusion matrix in Fig. 7 reveals the model’s performance on the test dataset, allowing for a comparison with the previously discussed validation matrix. Overall, the model’s accuracy on the test set is comparable to but slightly lower than the validation set, obtaining a precision of 87.2%. The class results are slightly lower for most classes except for Forest and Water, where accuracy is slightly higher. Grass shows an accuracy of 81.77% in the test compared to 88.83% in the validation, with a notable increase in confusion with Scrubland and Others. Scrubland has an accuracy of 83.67% in the test versus 88.16% in the validation, with a significant increase in confusion with Grass and Forest. Forest, on the other hand, improves in the test with an accuracy of 92.03% compared to 89.83% in the validation, although confusion with Scrubland is higher. Others show an accuracy of 92.52% in the test, improving from 89.95% in the validation, with less overall confusion. Water has the highest accuracy in both matrices, with 96.30% in the test versus 94.76% in the validation, with minimal errors.

Although the model demonstrates robust overall performance, the decrease in accuracy for Grass and Scrubland classes in the test set, along with increased mutual confusion, points to areas for potential improvement. It is important to note that the labelling of the dataset was primarily performed through a semi-automated approach, grouping pixels based on their homogeneity using the RGB values, rather than at the pixel level. However, this testing process relied on photo interpretation, which, as a more detailed and exhaustive validation method, likely introduces more errors, especially for visually similar classes. This increased level of scrutiny could explain the observed drop in accuracy for these classes, as small differences in appearance may result in misclassifications. Additionally, the test dataset was independent and not seen by the model during training, meaning that the model was evaluating its performance on new, unseen data. This introduces additional challenges, particularly when working with classes that share similar characteristics or exhibit high temporal and spatial variability. The testing area was also significantly larger and geographically diverse compared to the training area, contributing further to the variability in the Grass and Scrubland classes.

## Discussion

The evolution of land cover classification methodologies reflects significant advancements in remote sensing technologies, computational algorithms, and standardization frameworks. Contemporary approaches integrate multisensor data fusion, deep learning models, and modular classification systems to address historical challenges in spectral confusion, intra-class variability, and regional harmonization. Recent studies, such as those by Irwin et al. (2017) and Shakya et al. (2023), demonstrate that fusion-based methods combining synthetic aperture radar (SAR), light detection and ranging (LiDAR), and optical imagery improve classification accuracy compared to conventional techniques. Standardization frameworks like the Land Cover Classification System (LCCS) in Di Gregorio (2005), which offers a standardized approach to land cover mapping, promote global interoperability while accommodating local ecological gradients and nuances. Despite the increasing sophistication of these methodologies, photo interpretation and traditional data augmentation techniques remain essential in generating datasets for the use of these more advanced techniques. Data augmentation methods enhance dataset size and variability, allowing models to generalize better without requiring extensive ground truth data. Additionally, modular classification systems increasingly integrate very-high-resolution (VHR) RGB imagery with lower-resolution multisensor data, such as Sentinel imagery, to balance spatial detail with spectral richness. Moreover, when classifying large areas, it is crucial to rely on lower-resolution data such as Sentinel, given the high cost of acquiring drone-based multispectral imagery at resolutions comparable to RGB.

Although technology has advanced significantly, a solid photo-interpreted dataset is still required to begin the work, which involves a high temporal and computational cost. For this reason, data augmentation techniques remain essential for expanding the size and variability of datasets, improving the generalization ability of models without the need for large amounts of reference data. Our study confirms that conventional data augmentation techniques are still valuable and highlights the ones that contribute the most in land cover classification tasks. These techniques have proven to be especially effective when working with limited datasets. Although deep learning models (DLMs) have revolutionized land cover mapping, particularly regarding the scarcity of data in very high-resolution (VHR) images, challenges related to model scalability and label consistency still persist. Recently, models like the Segment Anything Model (SAM) in the study Kirillov et al. (2023) have emerged, aimed at transforming image labelling using a zero-shot approach that facilitates the annotator’s task of labelling the initial dataset. This reduces the annotation burden in resource-poor environments. However, SAM’s performance in high-resolution rural landscapes remains an underexplored area. On the other hand, as seen in our study, this approach should not replace data augmentation but rather complement it.

Our study highlights the efficacy of data augmentation techniques in improving land use and land cover (LULC) classification in very high-resolution images (25 cm), particularly when working with limited datasets. The applied augmentation techniques, both radiometric and geometric, not only increased the variability of the training set but also enhanced the generalization capability of the models. This finding aligns with previous research that underscores the positive impact of data augmentation on remote sensing image classification, such as the work by Stivaktakis et al. (2019) and Shorten and Khoshgoftaar (2019), who explore various data augmentation techniques independently. However, unlike these studies, our work evaluates combinations of geometric and radiometric transformations, allowing for the exploration of additional synergies to improve model generalization.

Our study also builds on the research by Du et al. (2021), who used only the DeepLabv3 + architecture—an approach we also explored and found to give the best results in validation. Unlike their work, which uses object-based image analysis (OBIA) to classify homogeneous image segments, our study focuses on pixel-level classification, aiming to address the limitations of smaller datasets using data augmentation techniques. While Du et al. worked with a much larger dataset (21 km^2^ vs. 1.3 km^2^ in our case), our work addresses the complexities posed by highly heterogeneous vegetation classes, making segmentation more challenging.

Additionally, Hao et al. (2023) review a wide range of data augmentation techniques but do not include combinations of radiometric transformations, such as those evaluated in our study. We highlight the importance of combining radiometric and geometric transformations to improve generalization in dynamic and heterogeneous environments, especially in cases where lighting and acquisition conditions vary. Our detailed analysis also reveals that applying multiple transformations simultaneously does not always result in better performance. In fact, when all techniques were used together, performance decreased, likely due to the noise added to the training set. This finding aligns with studies such as Yang et al. (2022), which warn about the potential negative effects of excessive transformation on model learning.

Despite these advancements, limitations were identified in the classification of vegetation classes such as grasses and shrubs, which showed greater confusion due to their high temporal and spatial variability. This phenomenon highlights the need to expand training datasets with more representative and diverse samples. The incorporation of additional spectral data, such as NIR bands, along with topographic information derived from elevation, has proven to be an effective tool for improving discrimination between vegetation classes, addressing one of the main limitations of our study, as demonstrated in Garioud et al. (2022). This work explores advanced techniques for semantic segmentation in highly variable environments, successfully classifying more diverse vegetation, including conifers, deciduous trees, shrubs, vineyards, and herbaceous vegetation, and highlighting the usefulness of multispectral bands for this purpose. Additionally, it evaluates the impact of data augmentation techniques on performance improvement, albeit not as pronounced as in our case. This is because, when working with a significantly larger base dataset, these techniques, although effective, do not have as marked an impact as observed in our study, where the limited size of the dataset highlights their importance.

Our study makes a significant contribution by being the first to address the impact of data augmentation techniques and their combinations on land cover classification tasks, an area that had not been investigated in detail in the existing literature. Our results show that data augmentation techniques are crucial for improving accuracy in limited datasets, achieving substantial improvements in result quality. While there are methods that facilitate initial labelling through photo interpretation, this process remains laborious and costly. However, by applying controlled data augmentation, it is possible to enrich the dataset without compromising result quality and with a smaller size of the photo-interpreted dataset. The combination of this approach with Sentinel images, which are updated every 5 days, allows us to multiply the data quantity without affecting accuracy, as land cover changes typically do not occur within such a short interval. This hybrid approach, which integrates very high-resolution images with Sentinel data, leverages the temporal variable provided by the satellite. This approach complements previous studies, such as Garioud et al. (2023), which demonstrate the effectiveness of hybrid approaches for land cover classification tasks.

## Conclusions

This study successfully demonstrates the efficacy of data augmentation techniques in enhancing land cover classification using deep learning models, particularly when working with limited ultra-high-resolution (25 cm) datasets. Through a systematic evaluation of various augmentation strategies, the research provides valuable insights for researchers and practitioners in the fields of remote sensing and land cover mapping.

A key conclusion from the study is that data augmentation significantly improves mode performance, with some cases showing improvements of up to 30%. This highlights the importance of augmentation techniques in addressing the challenges posed by limited training data in high-resolution land cover classification tasks.

Among the strategies tested, the combination of flip, contrast, and brightness adjustments emerged as the most effective. When applied to the DeepLabV3 + architecture, this optimized model achieved an impressive accuracy of 0.89 and an IoU of 0.78, setting a new benchmark for and cover classification using limited data.

The study also offers a practical framework for identifying the most effective augmentation strategies, potentially saving considerable time and resources for future land cover classification projects. The successful application of the optimized model to generate land cover maps for multiple years (2014, 2017, and 2019), with high accuracy (87.2%), demonstrates the robustness and transferability of the approach across temporal datasets.

However, the research also reveals that excessive data augmentation can lead to diminishing returns or even a decrease in performance. This underscores the need for careful selection and combination of augmentation techniques to avoid overfitting or degrading model effectiveness.

Future research could explore the integration of multispectral data, such as NIR bands, and topographic variables to further enhance the classification of complex vegetation classes. The addition of NIR bands could provide improved discrimination of vegetation types by leveraging their sensitivity to chlorophyll content, while topographic variables such as slope and elevation could account for environmental factors influencing vegetation distribution. These advancements could address current limitations and further improve classification accuracy, particularly for highly heterogeneous and dynamic environments.

Finally, the study addresses a critical gap in the literature by providing a comprehensive and comparative evaluation of data augmentation methods specifically applied to land cover classification. It offers valuable guidance for future studies in this domain and contributes significantly to the field of high-resolution land cover classification.

## Data Availability

No datasets were generated or analysed during the current study.
